# Clinical review of alkalization therapy in cancer treatment

**DOI:** 10.3389/fonc.2022.1003588

**Published:** 2022-09-14

**Authors:** Reo Hamaguchi, Masahide Isowa, Ryoko Narui, Hiromasa Morikawa, Hiromi Wada

**Affiliations:** Japanese Society on Inflammation and Metabolism in Cancer, Kyoto, Japan

**Keywords:** cancer, cancer metabolism, tumor microenvironment, alkalization therapy, urine pH, chemotherapy

## Abstract

One of the most unique characteristics of cancer metabolism is activated aerobic glycolysis, which is called the “Warburg effect”, and is a hallmark of cancer. An acidic tumor microenvironment (TME) resulting from activated anaerobic glycolysis is associated with cancer progression, multi-drug resistance, and immune escape. Several *in vitro* and *in vivo* studies reported that neutralization of the acidic TME by alkalizing agents, such as bicarbonate, resulted in the suppression of cancer progression and a potential benefit for anti-cancer drug responses. In clinical settings, alkalizing effects were achieved not only by alkalizing agents, but also by a following a particular diet. An epidemiological study demonstrated that more fruits and vegetables and less meat and dairy products are associated with an increase in urine pH, which may reflect the alkalizing effect on the body. However, it remains unclear whether alkaline dietary intervention improves the effects of cancer treatment. Moreover, there are few clinical reports to date regarding cancer treatments being performed on patients together with alkalization therapy. In this review, we investigated whether alkalization therapy, which includes an alkaline diet and/or alkalizing agents, improves cancer treatment.

## Introduction

There are numerous lines of evidence that pH gradient reversal, intracellular alkalization, and extracellular acidification are commonly seen in malignant tumors and are associated with the progression, metastasis, and multidrug resistance (MDR) of cancer cells (1–3). Activation of aerobic glycolysis, which is also known as the “Warburg effect”, is a characteristic feature of cancer metabolism and a hallmark of cancer (4). Cancer cells require rapid adenosine triphosphate (ATP) generation to maintain their energy state, increase macromolecule biosynthesis, and maintain an appropriate cellular redox state for their survival and growth. Activated aerobic glycolysis produces reduced nicotinamide adenine dinucleotide phosphate, which is necessary to maintain redox balance, and also acts as an antioxidant to protect against reactive oxygen species that are generated during rapid cancer growth (5). Therefore, aerobic glycolysis, which is a shift from ATP generation by oxidative phosphorylation to ATP generation by glycolysis, is observed even under normal oxygen concentrations (5–7). The constant increase in aerobic glycolysis is considered to be an adaptation to the hypoxia that occurs as precancerous lesions become increasingly distant from the blood supply (6). However, recent reports indicate that the glycolytic phenotype is an important component of the metabolic reprogramming of cancer cells that occurs early in carcinogenesis, i.e., before the development of tissue hypoxia (1, 5–7). Aerobic glycolysis can be caused by genetic instability, mutations, abnormal gene expression, or altered signaling pathways (1). Increased lactate production owing to increased glycolysis leads to acidosis of the extracellular tumor microenvironment (TME) (5, 6, 8). Moreover, the systemic extrusion of H^+^ by different proton transporters, and the neutralization of protons in cancer cells by bicarbonate anions from the chloride bicarbonate exchanger are the main mechanism for reversing the pH gradient in cancer cells (5, 7, 9, 10). The extrusion of H^+^ from cancer cells is positively regulated by several membrane-bound proton transporters, such as Na^+^/H^+^ exchanger 1 (NHE1), Na^+^/K^+^ ATPase pump, vacuolar H^+^-ATPase (V-ATPase), H^+^/Cl^−^ symporter, monocarboxylate transporter (MCT), and carbonic anhydrase (CA) (10).

Although emerging lines of evidence from both *in vivo* and *in vitro* studies suggest that the reversed pH gradient of cancer cells may be a promising new target of cancer treatment, the mainstream treatments for advanced cancer are chemotherapeutic drugs and molecularly targeted therapies, and there are few strategies aiming at the pH regulation of cancer cells in clinical settings. In this article, we aimed to summarize the association between the acidic TME and cancer treatments, and introduce several approaches of alkalizing the external TME and associated treatment strategies.

## An acidic TME leads to resistance to cancer therapy

A direct cause and effect association among the degree of MDR, decrease in external tumor pH (pHe), and increase in internal tumor pH (pHi) has been reported, and the reversed pH gradient of cancer cells is known as a key factor in driving the progression of malignancy and resistance to conventional therapies (8, 11, 12). An *in vitro* study of human lung tumor cells demonstrated that a close to 2,000-fold increase in doxorubicin resistance was observed when the pHi increases from 7.0 to 7.4 (13). Furthermore, a decrease in pHe and increase in pHi mediated by proton-extruding mechanisms is responsible for not only the maintenance of MDR but also protection against the induction of apoptosis (14–16). P-glycoprotein, a drug efflux transporter, is regulated in a pH-dependent manner, and a decrease in pH of the TME has the potential to enhance its efflux function (17, 18). Moreover, the uptake of weakly basic chemotherapeutic drugs by tumors is highly affected by the pH of the TME and the ionization properties of the drug (19). That is, an acidic TME reduces the cellular uptake of weakly basic chemotherapeutic drugs, such as anthracyclines (doxorubicin, daunorubicin, mitoxantrone, etc.) because weakly basic chemotherapeutic drugs become trapped in extracellular compartments owing to being positively charged in acidic conditions (20–22). Characteristics of the TME, such as having an acidic pH, being hypoxic, and lacking nutrients, are associated with cancer stem cells that demonstrate self-renewal and multilineage potential, leading to heterogeneity within the tumor and contributing to treatment resistance and clinical relapse (23). It is also known that the acidic TME is associated with a decreased anti-cancer immune response. Lactic acid in the TME suppresses immune cells, such as dendritic cells, natural killer cells, cytotoxic T cells, and macrophages, resulting in the inhibition of antitumor immune responses, and cancer immune escape (24, 25). An *in vitro* study demonstrated that the acidic TME is associated with both the suppression of T-cell responses and a decrease in the secretion of IFN–γ and TNF–α, and the effects of anti-programmed cell death 1 therapy were reported to be enhanced by alkalization using bicarbonate in mouse models of melanoma (26).

In summary, reversal of the pH gradient of the TME of cancer cells leads to MDR and reduced cancer immunity, resulting in resistance to cancer therapy. Current cancer treatment strategies do not consider pH changes in cancer and its association with sensitivity to drug therapies, and treatment approaches aiming at pH regulation of the TME may hence be a future therapeutic strategy.

## Approaches of alkalization of the acidic TME

**Figure 1:**
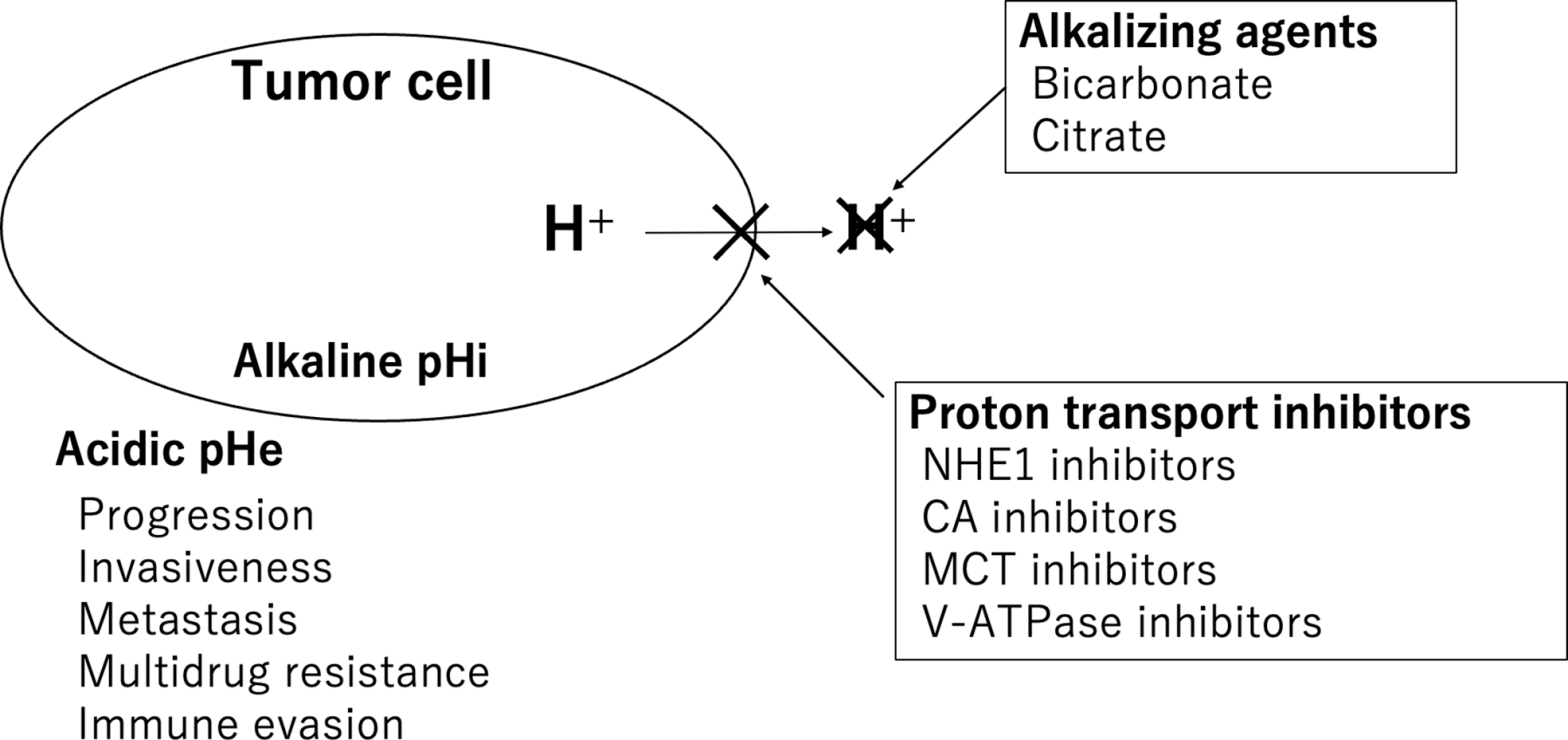
Therapeutic approaches targeting the acidic pH of the TME. Alkalizing agents and proton transporter inhibitors are shown.

There are two main therapeutic approaches that target the acidic pH of the TME. One is buffer therapy, in which alkalizing agents are administered to neutralize protons, and the other is the inhibition of proton efflux transporters expressed on the cancer cell membrane (**Figure 1**).

### Alkalizing agents

Several studies have been reported on buffer therapies that neutralize the acidic TME of cancer cells. Alkalizing agents, such as bicarbonate, are commonly used in *in vitro* and *in vivo* studies. A mathematical simulation study showed that oral bicarbonate consumption as a systemic pH buffer increases the pH of the external TME and inhibits tumor invasion (27). In mouse models of metastatic breast cancer, it was reported that bicarbonate administration increased the pH of the TME, resulting in the suppression of metastasis and improvements of survival rates (28). It was also reported that alkalization of the acidic TME improves the anticancer immune response. As described above, the effects of anti-programmed cell death 1 therapy in mouse models of melanoma have been shown to be enhanced by alkalization through bicarbonate consumption (26). A prospective clinical trial in healthy volunteers was conducted for investigation of the safety of the long-term consumption of sodium bicarbonate for cancer care, and demonstrated that 90 days of sodium bicarbonate consumption (median 0.17 g/kg/day) was feasible and safe, and an increase in urine pH as a surrogate marker for buffering effect was observed following bicarbonate intake (29). It has also been reported that the oral administration of sodium potassium citrate as an alkalizing agent increases $\text{HC}{\text{O}}_{3}^{-}$ concentrations in the blood and urine, leading to an increase in urine pH and neutralization of the acidic TME in a pancreatic cancer xenograft model, thereby enhancing the therapeutic effects of anticancer drugs (tegafur/gimeracil/oteracil) (30).

### Proton transport inhibitors

#### NHE1 inhibitors

NHE1 is known to play not only an essential role in the survival of normal cells, but also a key role in cancer progression. In normal cells, NHE1 is quiescent in the steady-state resting intracellular pH, and is activated only upon cytosolic acidification. In cancer cells, NHE1 is activated even at resting pH, and the activation of NHE1 directly results in an increase in intracellular pH and a decrease in extracellular pH of cancer cells (7). NHE1 is a major plasma membrane pump that extrudes intracellular protons from cells, and is associated with tumor growth and progression (7). There are several NHE1 inhibitors, including derivatives of amiloride, such as 5-(N-ethyl-N-isopropyl) amiloride, 5-(N,N-dimethyl) amiloride, 5-(N,N-hexamethylene) amiloride (HMA), and cariporide (9). *In vitro* and *in vivo* studies using breast cancer cells have reported that cariporide improves doxorubicin sensitivity (31). It was reported that a patient with metastatic ovarian cancer who was treated with amiloride as a Na^+^/H^+^ exchanger inhibitor showed a favorable outcome (32). However, as NHE1 is widely present in many tissues and plays a fundamental role in important physiological processes, there is a potential risk of life-threatening side effects associated with NHE1 inhibitors. To take advantage of NHE1 inhibition in cancer therapy, it will be important to develop drugs that selectively target NHE1 in tumors (33).

#### CA inhibitors

CA acts as a catalyst to reversibly hydrate carbon dioxide to produce bicarbonate and protons, and the overexpression of CA isoforms IX and XII is involved in cancer progression and metastasis (34). These enzymes contribute to acidification of the extracellular pH of cancer cells (35). Inhibitors of CA IX and CA XII are considered as potential anticancer agents, and several clinical trials using these inhibitors have been conducted (34). A study using girentuximab, a chimeric antibody against CA IX, was reported and showed no significant effects on recurrence-free survival in clear cell renal cell carcinoma. However, subgroup analysis showed that patients with high CA IX expression have significantly longer recurrence-free survival than those with low CA IX expression (36).

#### MCT inhibitors

The activated glycolysis of cancer cells results in the overproduction of lactate, which is transported out across the cancer cell membrane *via* the MCT (mainly MCT1) (9, 37). Expression of MCT1 and MCT4 has been reported to be a characteristic of cancer cells and to contribute to tumor invasiveness, and hence these MCTs are potential targets for cancer treatment (37). *In vivo* and *in vitro* studies on the effects of MCT1 inhibitors against diffuse large B-cell lymphoma and Burkitt lymphoma reported that the accumulation of intracellular lactate and cancer cell proliferation were reduced by these inhibitors (38).

#### V-ATPase inhibitors

V-ATPase is an ATP-dependent proton transporter that expels protons from cancer cells, and V-ATPase activation promotes the progression of cancer. The inhibition of V-ATPase was reported to reduce cancer cell growth and induce apoptosis in several *in vivo* and *in vitro* studies (39). Moreover, proton pump inhibitors (PPIs), which act as H^+^/K^+^-ATPases and are used for the treatment of gastric ulcers and gastroesophageal reflux, are also known to inhibit V-ATPase. *In vivo* and *in vitro* studies have shown that PPIs induce apoptotic cell death and lead to chemosensitization and reversal of chemoresistance *via* the inhibition of V-ATPase (40, 41). Population-based studies also reported that treatment with PPIs may prevent the progression of breast cancer (42, 43). Although clinical trials are limited, favorable results have been reported in three patients with advanced colorectal cancer treated with chemotherapy in combination with high-dose PPIs (44). In addition, in patients with metastatic breast cancer treated with a combination of chemotherapy and PPIs, significantly prolonged progression-free survival (PFS) and overall survival (OS) were observed compared with patients treated with chemotherapy alone (45).

## Can diet affect the pH regulation of the TME?

It is known that diet is associated with cancer risk. The World Cancer Research Fund/American Institute for Cancer Research reported their recommendations associated with food intake to reduce cancer risk as follows: ‘Eat a diet rich in wholegrains, vegetables, fruit and beans’ and ‘Limit consumption of red and processed meat’ (46). Although the benefit of an alkaline diet on cancer risk still remains unclear, a case-control study reported that a diet with a high acid load may increase lung cancer risk (47). However, to our knowledge, there are no studies to date regarding the association between food intake and pH of the TME. On the other hand, the acid-base load on the body can be affected by food. In a study investigating the effects of food on urine pH, the acid and base precursors in food were quantified and the potential renal acid load was calculated to predict net renal acid excretion, and the potential renal acid load of meat was calculated as +9.5 mEq, whereas that of fruit was −3.1 mEq and vegetables was −2.8 mEq (48). An epidemiological study showed that an alkaline diet consisting of high fruit and vegetable and low meat intake had a significant association with an increase in urine pH (49). Therefore, the alkalizing effect of food results in an increase in urine pH; however, further studies are required to clarify the association between an alkaline diet and pH of the TME.

## Clinical reports of alkalization therapy for cancer

Although pH regulation of the acidic TME is considered to be a potential target of cancer therapy, research on the effects of alkalizing agents and proton transport inhibitors on cancer are mainly limited to *in vivo* and *in vitro* studies, and there are few clinical reports regarding alkalization therapy for cancer treatment. In this section, we will introduce some retrospective studies of alkalization therapy for cancer conducted by our group.

First, we report on a retrospective study investigating the effects of an alkaline diet on advanced or recurrent non-small cell lung cancer patients with epidermal growth factor receptor (EGFR) mutations, who were treated with EGFR-tyrosine kinase inhibitor (TKI) (50). All patients in this study were given instructions to follow an alkaline diet as part of their routine clinical care. In this study, the mean urine pH (n = 11) was significantly increased after an alkaline diet, which was defined as that with a large amount of vegetables and fruits and minimal amount of meat and dairy products. Although the average dosage of EGFR-TKI administered to the patients was less than the standard dosage (56% ± 22% of the standard dosage), the median PFS was 19.5 (n = 11, range = 3.1–33.8) months. It is known that the median PFS reported in publications of a similar population treated with EGFR-TKI alone was 10.9–13.1 months (51, 52). This was a preliminary observational study that did not have a comparator group; however, the favorable results of these 11 cases might suggest the importance of the combination of alkalization and EGFR-TKI therapy.

**Figure 2:**
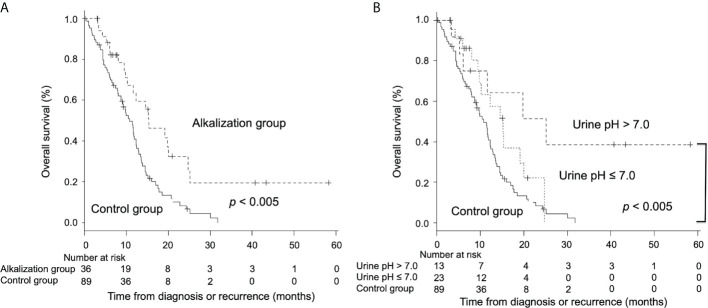
Overall survival between advanced pancreatic cancer patients who were treated with alkalization therapy plus chemotherapy and those who were treated with chemotherapy only. Kaplan–Meier curves of the OS of the alkalization group and the control group are shown. **(A)** The median OS of the alkalization group was significantly longer than that of the control group. **(B)** In patients with an increased urine pH (pH > 7.0), a more prolonged median OS was observed than in the control group. [Adapted from reference (54)].

Second, a retrospective study was conducted to investigate the effects of alkalization therapy performed concurrently with chemotherapy on recurrent or metastatic pancreatic cancer patients (53). A total of 28 patients with advanced pancreatic cancer who agreed to receive alkalization therapy, were treated with alkalization therapy, consisting of an alkaline diet with oral sodium bicarbonate (3.0−5.0 g/day). We found that alkalization therapy significantly increased the mean urine pH. A significantly prolonged median OS was observed in patients with a urine pH of higher than 7.0, compared with patients with a urine pH of 7.0 or lower (n = 28, 16.1 vs. 4.7 months; *p*< 0.05). Moreover, a retrospective case-control study was conducted to investigate the effects of alkalization therapy on chemotherapy outcomes in recurrent or metastatic pancreatic cancer patients (54). Patients in the alkalization group (alkalization therapy plus chemotherapy, n = 36), which included patients from the above retrospective study, were compared with patients in the control group (chemotherapy only, n = 89). The median OS was significantly longer in the alkalization group than in the control group (15.4 vs. 10.8 months; *p*< 0.005) (**Figure 2A**). In addition, the median OS of patients with an increased urine pH (pH > 7.0) in the alkalization group (n = 13) was significantly longer than that of the control group (n = 89) (25.1 vs. 10.8 months; *p*< 0.005) (**Figure 2B**). These studies suggest that alkalization therapy may be associated with more favorable outcomes in advanced pancreatic cancer patients treated with chemotherapy. A prospective randomized study is required in the future to clarify the effects of alkalization therapy.

**Figure 3:**
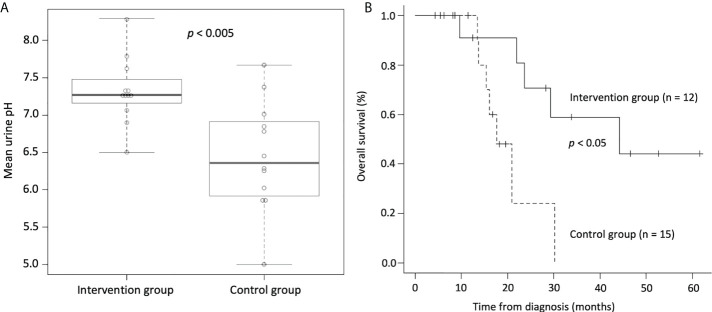
Urine pH and overall survival of small cell lung cancer patients who were treated with alkalization therapy plus vitamin C treatment together with chemotherapy and those who were treated with chemotherapy only. **(A)** Box-whisker plots of urine pH of the intervention group (alkalization therapy plus vitamin C treatment together with chemotherapy) and of the control group (chemotherapy only) are shown. Urine pH in the intervention group was significantly higher than that in the control group. The thick lines indicate the median values, the error bars indicate the maximum and minimum values, and the boxes indicate the values between the upper and the lower quartiles. **(B)** Kaplan–Meier curves of the OS of the intervention group and the control group are shown. The median OS of the intervention group was significantly longer than that of the control group. [Adapted from reference (55)].

Third, we conducted a retrospective study investigating the effects of alkalization therapy combined with intravenous vitamin C treatment on small cell lung cancer patients treated with chemotherapy (55). Twelve patients who agreed to be assigned to the intervention group (alkalization therapy plus vitamin C treatment together with chemotherapy) were compared with 15 patients in the control group (chemotherapy only) who did not agree to receive interventional treatment. Similar to our previous studies, urine pH of the intervention group was significantly increased compared with that of the control group (**Figure 3A**). A prolonged median OS was observed in the intervention group compared with the control group (44.2 vs. 17.7 months; *p*< 0.05) (**Figure 3B**). Although this study was a retrospective study with a small number of patients, alkalization therapy may be associated with favorable outcomes in patients with small cell lung cancer receiving chemotherapy, and it is speculated that supplementary intravenous vitamin C may have also affected their treatment outcomes. However, the effect of intravenous vitamin C treatment in combination with alkalization therapy remains unclear, and further investigation is needed.

As described above, we summarized our clinical studies of alkalization therapy, consisting of an alkaline diet and alkalizing agents, such as bicarbonate. Alkalization therapy can be used in conjunction with any of the current standard chemotherapies, and may improve the outcomes of standard chemotherapies. However, these studies were not randomized, and were retrospective studies that analyzed a small number of patients from a single center, and hence the results should be interpreted with caution. Moreover, these clinical studies focused on patients with non-small cell lung cancer, pancreatic cancer, and small cell lung cancer, and did not investigate patients with other cancer types. In addition, our group has encountered patients with renal cancer, malignant lymphoma, gastric cancer, and breast cancer in whom alkalization therapy increased their urine pH, which may have been associated with their favorable outcomes. However, these are only case reports and require further investigation (56).

It was reported that intestinal alkalization by bicarbonate treatment showed a preventive effect for irinotecan-induced diarrhea in both *in vivo* and *in vitro* studies (57). In clinical studies investigating whether oral administration of bicarbonate (1.8–2.0 g/day) has preventive effects for irinotecan-induced diarrhea in patients with non-small cell lung cancer, small cell lung cancer, and colorectal cancer, no significant differences were observed in the effects of chemotherapy between the bicarbonate-treated and non-treated groups (58, 59). However, the effects of bicarbonate administration as alkalization therapy requires further investigation, as the number of patients in these previous studies were also small, the amount of bicarbonate consumption was low, and urine pH was not measured. Thus, there are not enough clinical studies to date to validate the efficacy of alkalization therapy, and further studies focusing on the treatment of alkalizing agents or proton transport inhibitors are required to further clarify the effects of alkalization therapy.

## Future directions of alkalization therapy

Alkalization therapy is a buffering therapy aimed at neutralizing the acidic TME. An animal study has shown that there is a correlation between changes in pH of the TME and changes in urine pH induced by alkalizing agents (30). Alkalization therapy tended to be more effective in patients with a higher urine pH in our clinical studies described above (50, 53–55), suggesting that urine pH may be an alternative indicator of the pH around cancer cells. It should be noted that these studies have not demonstrated the association between urine pH and tumor pHe/pHi ratio. Blood pH is tightly regulated, and the $\text{HC}{\text{O}}_{3}^{-}$ buffer system plays an important role in maintaining blood pH homeostasis by balancing the composition of carbonic acid, $\text{HC}{\text{O}}_{3}^{-}$ and carbon dioxide. In addition, renal filtration regulates the blood concentration of $\text{HC}{\text{O}}_{3}^{-}$ through glomerular filtration and acid secretion (60). It is speculated that bicarbonate administration increases the blood $\text{HC}{\text{O}}_{3}^{-}$ concentration, delivering excess $\text{HC}{\text{O}}_{3}^{-}$ into the tumor, where $\text{HC}{\text{O}}_{3}^{-}$ molecules traps H^+^ ions in the TME and form carbonic acid, resulting in neutralization of the tumor pHe (28). However, further objective evaluation of the association between urine pH and pH of the TME is needed. One method of measuring pH in tumor tissue is ^31^P-magnetic resonance spectroscopy (^31^P-MRS). It has been reported that measurement of pH by MRS is largely standardized, providing an accuracy of ± 0.1 pH units (61). Novel imaging probes have been developed to assess the acidic TME. ^89^Zr-labeled pH-low insertion peptide is a radiopharmaceutical imaging probe for *in vivo* analysis to quantify the acidic TME using positron emission tomography, and has potential clinical applications (62). Acido-chemical exchange saturation transfer magnetic resonance imaging can measure the extracellular pH of the TME using the ratio of two pH-dependent signals, and may be useful in revealing the association between urine pH and pH of the TME (63, 64). It is also necessary to investigate how alkalizing therapy affects the expression of cancer-associated genes, and whether the response to alkalizing therapy differs depending on the gene expression status. In addition, as regulation of pH in the body is affected by daily diet and lifestyle, numerous factors are involved, and an exhaustive analysis using artificial intelligence may be useful in the future.

## Conclusions

We here summarized the therapeutic approaches against cancer targeting pH regulation. Although alkalization therapy as a buffer therapy using alkalizing agents, and therapies inhibiting proton transporters expressed on cancer cells are potentially promising, their clinical applications remain still limited. Further clinical investigations are hence needed in the future.

## Author contributions

RH performed the literature review and wrote the article. MI, RN, HM, and HW performed the literature review. All authors contributed to the article and approved the submitted version.

## Funding

This study did not receive any specific grants from funding agencies in the public, commercial, or not-for-profit sectors.

## Acknowledgments

The authors thank Dr. Helena Akiko Popiel of Tokyo Medical University for her editing of this article.

## Conflict of interest

The authors declare that the review was conducted in the absence of any commercial or financial relationships that could be construed as a potential conflict of interest.

## Publisher’s note

All claims expressed in this article are solely those of the authors and do not necessarily represent those of their affiliated organizations, or those of the publisher, the editors and the reviewers. Any product that may be evaluated in this article, or claim that may be made by its manufacturer, is not guaranteed or endorsed by the publisher.
